# Instrument for Producing Compression in Orchitis

**Published:** 1855-01

**Authors:** T. L. Ogier

**Affiliations:** Charlestown, S. C.


INSTRUMENT FOR PRODUCING COMPRESSION IN ORCHITIS. BY T. L. OGIER, M. D., OF CHARLESTOWN, S. C.—Messrs. Editors—I send you the following description of an invention of mine, which, if you think worthy, insert in your journal.

The treatment of orchitis by adhesive straps, applied so as to exercise a gentle pressure on the gland, is, I believe considered by surgeons, a great improvement to the old plan of leeching, fermentations, cold applications, &c.—the bandaging giving immediate relief from pain, and reducing the swelling considerably in a few hours, and this without confining the patient to his bed.

In treating cases of orchitis, I have found the swelling so much reduced the morning after the straps were applied, that they no longer gave any support to the testicle, and, therefore, to be efficient, had to be reapplied every ten or twelve hours, or oftener; for as soon as the pressure of the plaster is taken off of the gland, by the latter becoming smaller, the straps no longer act beneficially, and the curative process is arrested until a fresh support is given to the swelling by a reapplication of the dressing. Now, this constant reapplication of the straps is troublesome and painful, and if not attended to as soon as they become loose, no longer give any support to the enlarged gland, and the cure is protracted.

To remedy this defect, it seemed to me that an elastic pressure exerted gently on the swelling, would be efficient—a pressure that would be constantly exercised on the swelling, notwithstanding it became reduced in size. For this object I had made an oval bag, about the size of a turkey egg, netted of thin india-rubber thread —the body of this bag was netted with pretty coarse meshes, so as to be open and cool, but the neck was netted much closer and finer. When this is stretched open and the testicle put into it, the pressure, on account of the closer work (around the mouth of the bag) is a little greater than it is elsewhere—it therefore remains on perfectly well, and there is a gradual and gentle pressure exerted over the whole gland, until the swelling is completely reduced.—*Charleston Medical Journal and Review.*

